# Lyoprotectant Formulation and Optimization of the J-Aggregates Astaxanthin/BSA/Chitosan Nanosuspension

**DOI:** 10.3390/biom13030496

**Published:** 2023-03-08

**Authors:** Yingyuan Zhao, Zhaoxuan Wang, Shuxian Liu, Shiying Xie, Yinchun Xie, Ruifang Li, Hiroaki Oda

**Affiliations:** 1 College of Biological Engineering, Henan University of Technology, Zhengzhou 450001, China; 2 Graduate School of Bioagricultural Sciences, Nagoya University, Nagoya 464-8601, Japan; 3 School of Medicine and Pharmacy, Ocean University of China, Qingdao 266003, China

**Keywords:** lyophilization, astaxanthin nanoparticles, freeze-drying protectant

## Abstract

Astaxanthin is a carotenoid with excellent antioxidant activity. However, this small lipid-soluble molecule is insoluble in water and has low stability. Although this situation can be improved when astaxanthin is prepared as a nanosuspension, the aqueous form is still not as convenient and safe as the dry powder form for storage, transport, and use. The lyophilization process provides better protection for thermosensitive materials, but this leads to collapse and agglomeration between nanoparticles. To improve this situation, appropriate lyophilization protectants are needed to offer support between the nanoparticles, such as sugars, amino acids, and hydroxy alcohols. The purpose of this work is to screen lyophilization protectants by single-factor experiments and response surface optimization experiments and then explore the optimal ratio of compound lyophilization protectants, and finally, make excellent astaxanthin/BSA/chitosan nanosuspension (ABC-NPs) lyophilized powder. The work shows that the optimal ratio of the compounding lyophilization protectant is 0.46% oligomeric mannose, 0.44% maltose, and 0.05% sorbitol (*w*/*v*). The ABC-NPs lyophilized powder prepared under the above conditions had a re-soluble particle size of 472 nm, with a ratio of 1.32 to the particle size of the sample before lyophilization. The lyophilized powder was all in the form of a pink layer. The sample was fluffy and dissolved entirely within 10 s by shaking with water. Consequently, it is expected to solve the problem of inconvenient storage and transportation of aqueous drugs and to expand the application of nanomedicine powders and tablets.

## 1. Introduction

Astaxanthin is a carotenoid with the chemical formula C_40_H_52_O_4_, which has a β-viologen ring at each end and can scavenge free radicals to act as an antioxidant [1,2]. Astaxanthin is mainly derived from *Haematococcus pluvialis* [3,4]. It has many functions, such as anti-inflammatory, vision protection, anti-aging, immunity enhancement, anti-tumor, and maintenance of the central nervous system [5,6,7]. Lu et al. [8] reported that hydrophobic astaxanthin monomer molecules can aggregate in hydrated solvents, resulting in two significantly different aggregates. The color, structure, optical properties, and physiological activity of the various aggregates can be dramatically different [9]. There are two forms of aggregation, the card-pack structure for H-type aggregates, and the head–tail structure for J-type aggregates. In this case, the card-pack system is composed of astaxanthin monomers arranged face-to-face in parallel. The head-to-tail system comprises of astaxanthin monomers arranged in parallel and staggered manner [9,10]. However, astaxanthin is a lipid-soluble active small molecule. Its water solubility and stability are poor [11].

In this study, we selected whey protein and chitosan as nanocarrier materials. We then used molecular self-assembly and spin evaporation techniques to encapsulate astaxanthin in a core-shell structure formed by whey protein and chitosan, resulting in the preparation of astaxanthin/bovine serum albumin/chitosan nanoparticles (ABC-NPs). This method increased the stability of astaxanthin and improved its bioavailability of astaxanthin. Chitosan [12] is a product of natural chitin deacetylation linked by β-(1→4) glycosidic bonds. It is the only polysaccharide in nature that contains a free amino group. In addition, chitosan is safe, non-toxic, degradable, and biocompatible. Whey protein [13] is a valuable protein extracted from milk. It contains α-lactalbumin (α-La), β-lactoglobulin (β-Lg), and bovine serum albumin (BSA), which can bind to a variety of cations, anions, and other small molecules, and thus has a variety of activities. In addition, it has high nutritional value and is easily digested and absorbed, so it is a suitable carrier for drugs. In a specific ratio of water and organic solution, astaxanthin small molecules spontaneously self-aggregate to form aggregates. They are present in the hydrophobic micro-regions of nanocarriers constructed from chitosan and whey protein.

As aqueous solutions, nanosuspensions are not as convenient and safe to store, transport, and use as dry powders. Freeze drying allows better preservation of heat-sensitive materials, such as the material’s color, structure, and chemical properties. However, freeze-drying is the process of removing inter-particle moisture, which leads to the collapse and aggregation of the nanocomposites. In addition, the proteins on the surface of the nanoparticles may also undergo structural changes due to mechanical forces [14]. Appropriate lyophilization protectants can provide support in ABC-NPs, such as sugars, hydroxy alcohols, amino acids, and monosaccharide mixtures [15,16]. Among them, sugars are classified as monosaccharides, disaccharides, and glycans. Monosaccharides include glucose; disaccharides include maltose and lactose; oligosaccharides include oligomannose and oligomaltose; alcohols include mannitol, oligomannitol, and sorbitol; amino acids include glycine, lysine, and glutamic acid [17]; mixed lyophilization protectants are obtained from the compounding of sugars and alcohols or sugars and amino acids [18]. In general, there are two theories to explain the mechanism of action of lyophilization protectants, namely the water substitution hypothesis and the glassy state hypothesis. Andreani et al. [19] explained the water substitution hypothesis as the interaction between sugar molecules and nanoparticles. They suggested that the abundant hydroxyl groups in sugar molecules can form hydrogen bonds with sugars and proteins on the nanoparticle surface. In the case of dehydration, sugar can replace water molecules to interact with the nanoparticle surface and inhibit the dehydration-induced shortening of the distance between adjacent nanoparticles, thus stabilizing the nanosuspension system. The glassy state is the state of existence of a substance under certain conditions. In the glassy state, the substance exists in a non-crystalline form, the viscosity of the solution increases, the diffusion coefficient of the solute decreases, and crystallization is not easily formed. In the drying process, with the reduction of water, the concentration of the solution will increase significantly [20]. If the highly concentrated protectant solution does not crystallize, the mixture of protectant and water will be glassy and form a glassy state. This highly viscous glassy state surrounds the protein, creating a glassy carbohydrate. The movement of non-covalently bonded chains of proteins and biomolecules in glassy solutions is prevented, thus preserving the original conformation and physicochemical properties of the proteins and protecting the architecture of nanoparticles [21].

In this study, we selected six effective lyophilization protectants: oligosaccharides, chitosan, maltose, mannitol, sorbitol, and L-lysine. Sucrose has been used as a lyophilized protective agent for many years, but in recent years it has been found that sucrose metabolizes to fructose and produces potential cardiovascular toxicity, so this option is ruled out in this study [22]. The ABC-NPs complex was used as a model for the nanocarriers, and the water molecules in the complex were purged using a freeze-drying technique. Various characterizations of the lyophilized powders were used as evaluation criteria, such as particle size, polydispersity index (PDI), and re-solubilization time [23]. Then, three of the six lyophilization protectants were selected and compounded. Response surface analysis method was used to explore the optimal ratio of lyophilized protective agents. The above study is expected to solve the problem of inconvenient storage and transportation of ABC-NPs nanosuspension and expand the application of nanomedicine powder and tablet.

## 2. Materials and Methods

### 2.1. Materials

Chitosan (degree of deacetylation 90.25%) and chitooligosaccharides (degree of deacetylation 91.10%) were purchased from Zhejiang Aoxing Biotechnology Co., Ltd. (Zhejiang, China). Bovine serum protein (purity 97%) was obtained from Beijing Solarbio Technology Co. Astaxanthin (purity 99%) was purchased from Aladdin Biochemical Technology (Shanghai, China). Sorbitol, mannose, L/D lysine, and lactose were purchased from Solarbio, Beijing, China (Beijing, China). Oligomannose and oligomaltose were purchased from Jiangsu Duoyang Bioengineering Technology Co. Ltd. (Jiangsu, China). Sodium hydroxide (purity 99.7%), hydrochloric acid (purity 99.7%), and anhydrous ethanol (purity 99.7%) were purchased from Sinopharm Chemical Reagents Co. Ltd. (Beijing, China).

### 2.2. Fabrication of J Aggregates—Astaxanthin/Bovine Serum Albumin/Chitosan Nanoparticles

Dissolve 1000 mg of chitosan in 50 mL of sterilized ultrapure water, then add 3 mL of hydrochloric acid solution with pH 1 (concentration 0.1 mol/mL) and stir magnetically for one hour at room temperature. After the chitosan powder was fully hydrated, the hydrochloric acid solution with a concentration of 0.05 mol/mL was added dropwise and stirred until the chitosan solution was clear and transparent. Subsequently, sodium hydroxide solution with pH 13 was added to the chitosan solution to adjust its pH to 5–6. Finally, sterilized ultrapure water was added to the chitosan solution and fixed the volume at 200 mL. The chitosan stock solution with a concentration of 5 mg/mL was finally obtained. Dissolve 3 mg of astaxanthin in 100 mL of anhydrous ethanol and magnetic stirring for 25 min at 25 °C and protect it from light to obtain 0.03 mg/mL of astaxanthin ethanol solution. Accurately weigh 10 mg of bovine serum albumin and dissolve it in 100 mL of sterile ultrapure water. After stirring magnetically for 20–30 min at 25 °C, a solution of bovine serum albumin at a concentration of 0.1 mg/mL was obtained. After preparing the above three solutions, store them in a refrigerator at 4 °C. The ABC-NPs were prepared following the procedure described by our research team with some optimization [24]. Under light-proof conditions, we slowly poured 50 mL of astaxanthin solution with a concentration of 0.03 mg/mL into 50 mL of chitosan solution with a concentration of 0.1 mg/mL at an addition rate of 43 mL/min for about 70 s, followed by magnetic stirring for 20 min. Then 50 mL of bovine serum albumin solution with a concentration of 0.1 mg/mL was slowly added to the above-mixed solution with a spiking flow rate of 30 mL/min for 100 s, followed by magnetic stirring for 20 min. The rotary evaporator was turned on, the water bath was set at 35 °C, and the solution was rotated at 15 rpm for 70 min. When the mixture was evaporated to approximately 60 mL, we stopped the rotary evaporator, from which J Aggregates—astaxanthin/bovine serum albumin/chitosan nanoparticles, referred to as ABC-NPs, were prepared and stored in a refrigerator at 4 °C.

### 2.3. Determination of Particle Size and Zeta—Potential of ABC-NPs

The Malvern Nano ZS90 dynamic light scattering (DLS) instrument was chosen to measure the mean particle size, PDI, and potential of the ABC-NPs. Freshly prepared 1 mL ABC-NPs was added to the cuvette and placed in the sample chamber with an equilibration time of 120 s. The dispersant medium was water with a dispersant RI of 1.330 and a material RI of 1.45. Each sample was measured for three times and analyzed the average value [24].

### 2.4. Freeze-Drying Parameters of ABC-NPs

The bulk-prepared ABC-NPs were divided into seven vials of 2 cm diameter, of which all seven vials were mixed with 2.5 mL of ABC-NPs and 0.5 mL of different concentrations of lyophilized protectant so that the final concentrations of the oligomaltose solution, chitosan solution, mannitol solution, and sorbitol solution were 0.005%, 0.025%, 0.05%, 0.25%, 0.25%, and 0.5% (*w*/*v*) so that the final concentrations of maltose solution were 0.05%, 0.25%, 0.5%, 1%, and 2%, and so that the final concentrations of L-lysine solution were 0.0001%, 0.0002%, 0.001%, 0.005%, and 0.025%. The other two vials were 2.5 mL of ABC-NPs and 0.5 mL of sterile water mixture, both as blank control samples. The height of the sample in the vial is about 1 cm. After dispensing, they were sealed with a sealing film and rubber band tied with air permeability holes and put into a −80 °C refrigerator for 12 h of pre-freeze drying and then put into a freeze dryer (World Bank Technology Co., Ltd.) for 24 h of main freeze drying with a pressure of 0.03 MPa.

### 2.5. Observation of the Appearance of ABC-NPs Lyophilized Powder

Freshly prepared lyophilized powder of ABC-NPs was removed from the freeze dryer. Then, the samples were immediately photographed within 20 min. Subsequently, the situation of lyophilized powder and lyophilized powder wers observed after re-dissolution and the Tyndall effect.

### 2.6. Resolubilisation of ABC-NPs Lyophilized Powder

The prepared ABC-NPs lyophilized powder was re-dissolved with sterilized ultrapure water within 30 min. Then, researcher shook the lyophilized powder by hand and recorded the time to completely dissolve in the solid state. The particle size and the polydispersity index PDI (see Section 2.3 the lyophilized ABC-NPs lyophilized powder were measured by Malvern Nano ZS90 within one hour after the re-dissolution and recorded as re-dissolution particle size S_a_. Finally, we evaluated the amount of protective agent added and the corresponding effect.

### 2.7. Single-Factor Experimental Screening

According to the references [25] and the results of the preliminary experiments, the following six lyophilization protectants, which are L-lysine [26], maltose [27], oligomannose [28], chitosan [29], mannitol [30], and sorbitol [15] were selected. The final concentrations of oligomannose, chitosan, and mannitol lyophilized protectants were 0.005%, 0.025%, 0.05%, 0.25%, and 0.5% (*w*/*v*). The final concentrations of L-lysine were 0.0001%, 0.0002%, 0.0010%, 0.0050%, and 0.0250% (*w*/*v*). The concentration of lyophilization protectant can be defined as 0.5% (*w*/*v*) at 0.5 g/100 mL. Then the corresponding concentration of lyophilization protectant solution was prepared and added to ABC-NPs by the addition method. The ABC-NPs were prepared into lyophilized powder by freeze-drying procedure, and then the status and re-solubilization time of the lyophilized powder was observed and recorded. Finally, the particle size, potential, PDI, and particle size change ratio Sa/Sb (short for Size after/Size before freeze drying) of the lyophilized powder after re-solubilization were measured.

### 2.8. Single Lyophilisation Protectant Optimisation

In the above steps, six types of lyophilized protectants were screened firstly. Next, different lyophilized protectants were optimized one by one through controlling the material concentration and preparation conditions. Then the most suitable three lyophilized protectants were selected by using the state of the lyophilized powder and the particle size of the ABC-NPs complex after re-solubilization as evaluation criteria. Finally, the concentration continued to be refined.

### 2.9. Response Surface Optimization Experiments

Based on the single-factor experiments, an excellent compounded lyophilized protectant was prepaerd. The Box–Behnken experimental design with Design-Expert V8.0.6 software was used to establish a mathematical regression model to analyze the data using the particle size of ABC-NPs lyophilized powder as the evaluation index. A Box–Behnken design with three independent variables is shown in Table 1. Three independent variables were coded at three levels (−1, 0, 1) on the concentration of lyoprotectant, which produced an experimental design with 17-run experiments using a Design-Expert 8.0.6 [31].

All the experiments were conducted at random with the intent of minimizing the unexplained variability caused by systematic errors. A second-order polynomial equation was formed to study the effects of variables on the concentration of lyoprotectant. The equation indicates the effect of variables inline accordance with linear, quadratic, and cross-product terms:

$$Y=\beta_{0}+\sum_{i=1}^{3}\beta_{0}X_{i}+\sum_{i=1}^{3}\beta_{ii}X_{i}^{2}+\sum_{i=1}^{3}\sum_{j=i+1}^{2}\beta_{ij}X_{i}X_{j}$$

where *Y* is the concentration of lyoprotectant (%), *X_i_* and *X_j_* are the levels of variables, *β*_0_ is the constant term, *β_i_* is the coefficient of the linear terms, *β_ii_* is the coefficient of the quadratic terms, and *β_ij_* is the coefficient of the cross-product terms. Three-dimensional surface plots and contour plots were analyzed by the fitted polynomial equation. F-value and *p*-value were used to check the significance of the regression coefficient, while R^2^ and adjusted R^2^ were used to assess the model adequacy [31].

### 2.10. Analysis of Data

Experimental data were plotted for statistical analysis using Origin 9.0 and Excel, with different letters indicating significant differences (*p* < 0.05). Linear regression and ANOVA were performed on the data obtained from response surface tests using Design-Expert 8.0.6 software (the software is from Stat-Ease, Minneapolis, MN, USA).

### 2.11. Freeze-Drying Flow

**Figure 1:**
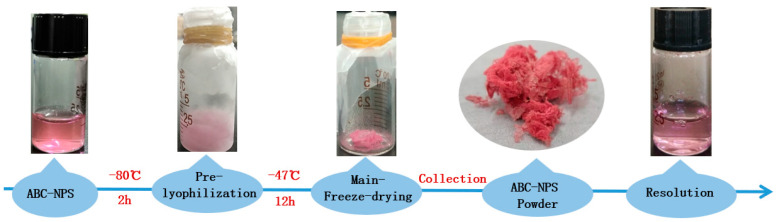
Freeze-drying flow chart.

The entire experimental process begins with the preparation of ABC-NPs in large quantities and then uses it as a backup storage solution (as shown in Figure 1). Follow-up experiments were conducted by taking some samples from a large batch of ABC-NPs. After ABC-NPs were removed, lyophilized protectants were added and then freeze-dried at −80 °C for 2 h and −47 °C for 12 h. Finally, the freeze-dried powder was re-dissolved, and the particle size, PDI, potential, and other data were measured.

## 3. Results and Discussion

### 3.1. Single-Factor Experiments

#### 3.1.1. Preparation of ABC-NPs

**Figure 2:**
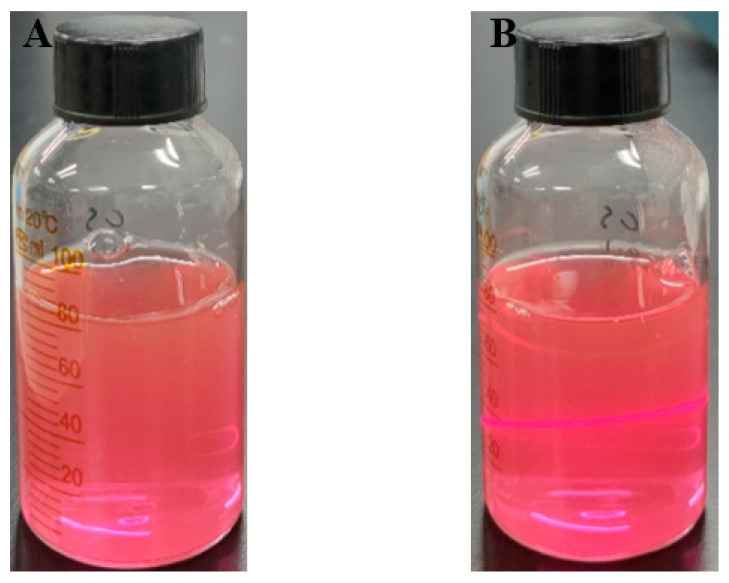
Bulk preparation of ABC-NPs. (**A**) is a digtial photograph of a bulk ABC-NPs nanosuspension; (**B**) is a Tyndal phenomenon of ABC-NPs.

The bulk astaxanthin/bovine serum albumin/chitosan nanoparticles (ABC-NPs) prepared according to the method in 2.4 are shown in Figure 2. The freshly prepared ABC-NPs with J-type astaxanthin aggregates nanosuspensionwas pink-purple and a clear Tyndal pathway could be observed by illumination with a red laser beam. The above phenomenon indicated that the ABC-NPs was well dispersed and homogeneous [24].

#### 3.1.2. Protection of Nanoparticles via Glycoconjugate Lyophilization Protectors

**Figure 3:**
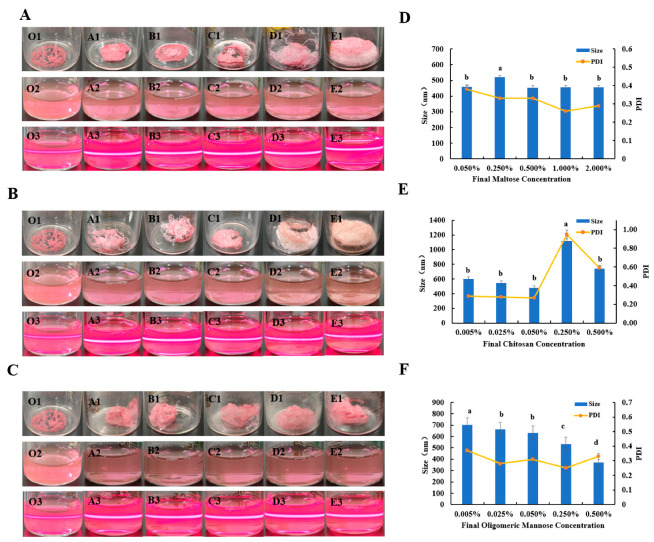
Effect of different concentrations of sugars (oligomeric mannose, chitosan, maltose) on ABC-NPs lyophilized powder: the appearance of ABC-NPs (lyophilized powder, re-soluble state, and Tyndall effect) (**A**–**C**) and particle size (**D**–**F**).

Following the method in 2.5, oligomeric mannose (0.005%, 0.025%, 0.05%, 0.25%, and 0.5% (*w*/*v*)), chitosan (0.005%, 0.025%, 0.05%, 0.25%, and 0.5% (*w*/*v*)), and maltose (0.05%, 0.25%, 0.5%, 1%, and 2% (*w*/*v*)) were added to the ABC-NPs nanosuspension at different concentrations. After freeze-drying, the prepared ABC-NPs lyophilized powder is shown as O1–E1 in Figure 3, and the O1, A1, B1, C1, D1, and E1 lyophilized powder samples in Figure 3A–C are all in skeletonized blocks, and sample E1 was in the state of adhesion. According to the method in 2.6, the samples were re-dissolved, and it was found that the re-dissolution time was gradually shortened as the concentration of sugar lyophilized protectant increased. All of them could be completely dissolved, and no pink granular precipitation was seen. The ABC-NPs after re-solubilization were irradiated with a laser pointer, as shown in Figure 3(O3–E3), and a clear red pathway appeared in almost all samples, except for the lyophilized powder at 1–2% chitosan concentration, which could not be completely re-solubilized, indicating good dispersion and homogeneity of the samples.

The particle size of the samples showed a gradual decrease with increasing oligomeric mannose concentration, especially at a concentration of 0.5 with the particle size reached 371 nm (Figure 3A,D), and Sa/Sb (short for Size after/Size before freeze drying) reached 1.02. However, all samples showed no clear trend in PDI. The values of PDI were below 0.4 and the potentials were almost always above +40 mV. The apparent situation indicated that the nanosuspension system was relatively stable. From Figure 3B, we observed that with the increase of chitosan concentration, the particle size and PDI both experienced a trend of decreasing and then increasing; the particle size and PDI became smaller and smaller when the chitosan concentration was 0.005%, 0.025%, and 0.05%. The smallest particle size reached 480 nm, the smallest PDI reached 0.27, the smallest Sa/Sb was 1.32, and the potentials are all above +40 mV. The above situation indicated that the nanosuspension system was relatively stable in the range of 0.005% to 0.05%. When the chitosan concentration was higher than 0.05%, both the particle size and PDI increased substantially and the potential decreased substantially, indicating that the system was unstable in this range. In addition, the particle size became smaller and then larger with increasing maltose concentration, and the PDI becomes smaller and smaller (Figure 3C,F). The smallest particle size was 454 nm at 0.5% maltose concentration, and the smallest Sa/Sb was 1.25. The smallest PDI was 0.30 at 0.005% maltose concentration, and the average potential was around +53 mV.

Sugars are the most commonly used freeze-drying protectants. Carbohydrate not only do not crystallize under normal conditions but also produce low eutectic mixtures or glassy substances with water molecules during the freezing process. Such substances prevent the growth of ice crystals and allow them to exist in amorphous or microcrystalline form, thus reducing the agglomeration and destruction of nanoparticles during lyophilization [32]. In addition, sugars contain a large number of hydroxyl groups, so when the protein-bound water is removed from the nanoparticle surface, sugars can replace the bound water and, thus, inhibit the conformational change of the protein [33,34].

#### 3.1.3. Protective Effect of Alcohols on Nanoparticles

**Figure 4:**
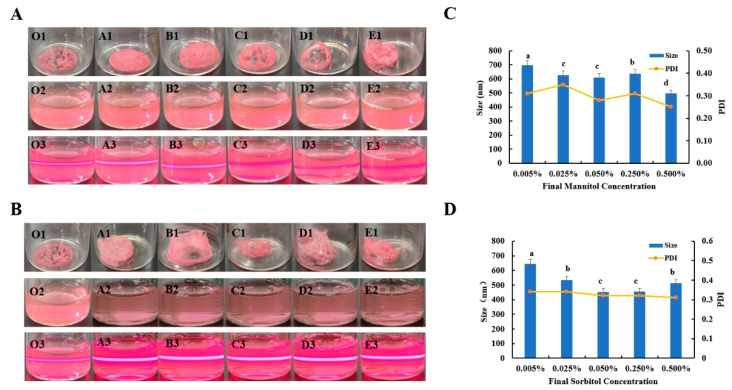
Effect of different concentrations of alcohols (mannitol, sorbitol) on ABC-NPs lyophilized powder: the appearance of ABC-NPs (lyophilized powder, re-soluble state, and Tyndall effect) (**A**,**B**) and particle size (**C**,**D**).

Following the method in 2.5, different concentrations of mannitol (0.005%, 0.025%, 0.05%, 0.25%, and 0.5% (*w*/*v*)) and sorbitol (0.005%, 0.025%, 0.05%, 0.25%, and 0.5% (*w*/*v*)) were added to the ABC-NPs nanosuspension. The above mixture was freeze-dried, and the prepared ABC-NPs lyophilized powder was shown as O1–E1 in Figure 4A,B below. As the concentration of mannitol increased, the state of the lyophilized powder in the blank control samples showed irregular dispersion. The four lyophilized powder samples marked as A1, B1, C1, and D1 in Figure 4A were in the form of loose powder lumps, while sample E was in the state of adhesion. When the samples were re-dissolved according to the method in 2.6, the dissolution rate of the samples gradually increased with the increase of mannitol concentration. However, as shown in O2–E2 in Figure 4A, the flocculent pink complex was still present in the sample with a mannitol concentration of 0.005%, even after shaking for more than 5 min. When the samples were irradiated with a laser beam, the status of the samples was shown as O3–E3 in Figure 4A, and a clear red pathway was visible in each sample, indicating good dispersion of all samples. Figure 4B indicated that the particle size gradually decreased with the increase of mannitol concentration, the PDI gradually decreased, and the minimum Sa/Sb reached 1.32.

The ABC-NPs lyophilized powder prepared according to the method in 2.5 was shown as O1–E1 in Figure 4B, but the four samples named O1, A1, B1, and C1 in Figure 4B were all irregularly shaped, while the two samples named D1 and E1 were in the form of adhesion. The samples were re-dissolved according to the method in 2.6, and the redissolution time was gradually reduced with the increase of sorbitol concentration. Finally, the samples were all completely dissolved, and no pink granular precipitation was seen. The samples were irradiated with the laser pointer, and the status of the samples is shown in Figure 4B(O3–E3). It can be seen that there is a clear red pathway in all samples, which indicates that the dispersion and homogeneity of the samples are good. Figure 4B exhibited that the particle size first gradually decreased and then gradually increased with the increase of sorbitol concentration. When the sorbitol concentration was 0.05%, the particle size was the smallest, reaching 454 nm, and the smallest Sa/Sb was 1.25. In addition, the PDI had a tendency to decrease gradually, and all of them are stable below 0.35. The above situation indicates that the samples have good homogeneity. The potentials are all above +40 mV, which indicates that the nanosuspensions are stable.

There are several reasons for the above situation. Polyhydric alcohols have a large number of hydroxyl groups in their structure. As the concentration of the protectant increases, the water molecules around the nanoparticles in suspension are replaced by the hydroxyl groups of mannitol, and the hydroxyl groups also interact with the hydrogen bonds in the protein molecules. The above situation keeps the protein structure on the surface of nanoparticles unchanged, avoiding agglomeration of nanoparticles and maintaining nanoscale stability [35]. Sorbitol is a low molecular weight, so it may adsorb back to the nanoparticle surface and protect them [15]. In addition, sorbitol has a shallow redox potential and can replace proteins that are oxidized first, and is, therefore, considered an antioxidant [34].

#### 3.1.4. Protective Effect of Amino Acids on Nanoparticles

**Figure 5:**
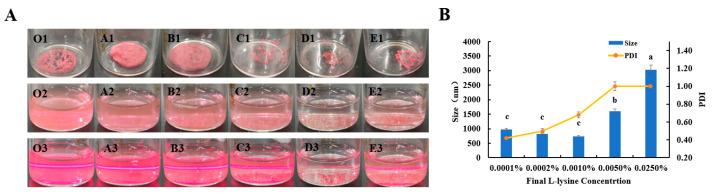
Effect of different concentrations of lysine on ABC-NPs lyophilized powder: the appearance of ABC-NPs (lyophilized powder, re-soluble state, and Tyndall effect) (**A**) and particle size (**B**).

The ABC-NPs lyophilized powder prepared according to the method in 2.5 is shown as O1–E1 in Figure 5A below. With the increase of the added L-lysine concentration (0.0001%, 0.0002%, 0.001%, 0.005%, and 0.025% (*w*/*v*)), these three lyophilized powder samples named O1, A1, and B1 in Figure 5A are powder blocks with a uniform skeleton. In contrast, the state of the lyophilized powder of the four samples called B1, C1, and D1 is irregularly scattered loose small particles, and sample E is noticeable loose gel-like particles. When the samples were re-dissolved according to the method in 2.6, the re-dissolution time was gradually extended as the concentration of L-lysine added increased, and even pink granular precipitates appeared at the bottom of the bottle for some samples. As shown in Figure 5A, the pink, coarse sediments in these four samples, named B2, C2, D2, and E2, increased with the increase of L-lysine concentration. The samples were irradiated with a laser pointer, and the status of the samples is shown in Figure 5A for O3–E3. There is a clear red pathway in only two samples, the blank sample, and sample A3, which indicates that the dispersion and homogeneity of the samples are good. However, the red pathways in the remaining four samples were unclear, indicating that the distribution and uniformity of the samples were relatively poor.

It can be seen from Figure 5B that the particle size decreases and the PDI increase with increasing concentration for the samples with L-lysine concentrations of 0.0001%, 0.0002%, and 0.001%. However, the particle size and PDI increased substantially after the L-lysine concentration reached 0.005%. The minimum Sa/Sb was only 2.24, and the maximum was 11.6, with a negative potential.

The studies of Kundu et al. [36,37] indicated that the positively charged side chain groups of lysine could interact with the groups of proteins to form spacers between nanoparticles, thus preventing nanoparticle agglomeration effectively. However, the study by Mohammed et al. [38] reported that the cryoprotection mechanism of lysine is biphasic (the cyroprotection profiles of the three amino acids tested were biphasic). As the concentration of amino acids increased, the lyophilization protection of amino acids showed first a positive and then a negative effect.. When the lysine:liposome concentration was 4:1, the vesicle size after lyophilization was comparable to the original size; when the lysine: liposome concentration was 10:1, it led to the aggregation of the vesicles, whose size increased significantly to 10 times the original size. Similar biphasic properties are also seen in the lyophilized cryoprotectants [38].

### 3.2. Box–Behnken Design Analysis

The particle size ratio Sa/Sb, the amount of lyophilization protectant, and the sample re-dissolution time can be used to screen the lyophilization protectant. Finally, three lyophilization protectants were selected from six types of lyophilization protectants, namely oligomeric mannose, maltose, and sorbitol. After re-dissolution, the minimum particle size ratios of the lyophilized powders prepared with the above-lyophilized protectants were 1.02, 1.25, and 1.25, respectively. According to the principle of lowest concentration to achieve the highest efficiency, after refinement of concentration, the optimal concentration range was selected as follows: the final concentration of oligomeric mannose was 0.3%, 0.4%, and 0.5% (*w*/*v*), and the final concentration of oligomeric mannose 0.4% (*w*/*v*) was selected as the center point of optimization; the final concentration of maltose was 0.4%, 0.5%, and 0.6% (*w*/*v*), and the final concentration of maltose 0.4% (*w*/*v*) as the center point of optimization; and final sorbitol concentrations of 0.15%, 0.25%, and 0.35% (*w*/*v*), and final sorbitol concentration of 0.25% (*w*/*v*) was selected as the center point of optimization.

#### 3.2.1. Statistical Analysis and the Model Fitting

Response surface is an effective optimization method when multiple factors affect the effect of lyophilization protectants on nanoparticle re-solubilization [39]. Based on single-factor experiments with the final concentrations of oligomeric mannose, maltose, and sorbitol as variables, a three-factor with three-level optimization experimental design was conducted. There were 17 runs for optimizing the three individual parameters, and the results are shown in Table 1. By applying multiple regression analysis, the relationship between response variables and the test variables was obtained as follows: Y = 445.80 − 8.63 × A − 21.5 × B + 0.13 × C + 5.00 × AB + 7.25 × AC − 2.00 × BC + 27.48 × A2 + 20.73 × B2 − 0.025 × C2. In this case, Y is the particle size of ABC-NPs after re-solubilization, and A, B, and C are the coding variables for oligomeric mannose, maltose, and sorbitol, respectively. We analyzed the regression equation, and the obtained ANOVA results were shown in Table 2. The *p*-value of the quadratic regression model was 0.0059, which was less than 0.01 (significant), while the lack-of-fit value was 0.7498, which was greater than 0.05 (not significant), indicating that the selection of the data model was reasonable; R^2^ (determination coefficient) and R^2^_Adj_ (adjusted determination coefficient) were 0.7990 and 0.9120, indicating a good fit of the quadratic regression equation to the test results. In addition, the lower C.V.% (coefficient of variation) is 2.49, which shows high reliability and accuracy. Based on the magnitude of the F-value, the strength of the effect of three key factors of lyophilized protectants on the particle size (variation) is maltose > oligomeric mannose > sorbitol. In summary, the model can analyze and predict the particle size change after nanoparticle lyophilization protectant addition and determine the optimal protectant compounding conditions.

#### 3.2.2. Analysis of Response Surface Plot

**Figure 6:**
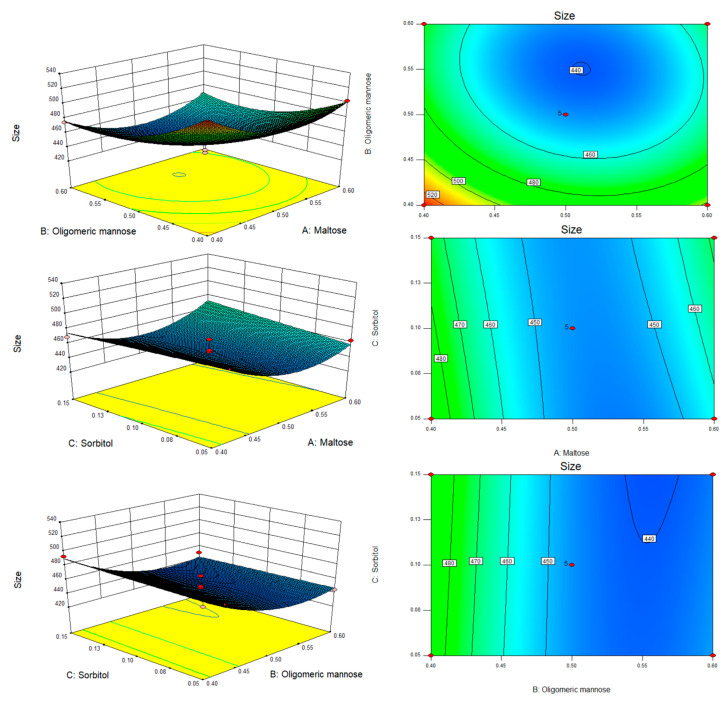
Response surface plots showing the effect of maltose (A), oligomeric mannose (B), and sorbitol (C) on the particle size of ABC-NPs.

The interactions between the optimal parameters and factors can be visualized from response surface analysis plots [40], while the shape of the contours can reflect the trend in the strength of the interactions between the factors [41]. Within the response surface methodology design, the interaction factors were AB, AC, and BC, and their response surfaces and contours are shown in Figure 6. The three-dimensional contour plots of oligomeric mannose concentration and maltose concentration are shown in Figure 6A. When the concentration of sorbitol is certain, the particle size of ABC-NPs shows a trend of increasing and then decreasing with the increase of oligomeric mannose and maltose concentrations. However, the interactions between oligomeric mannose and sorbitol and maltose and sorbitol were weak, as shown in Figure 6B,C.

### 3.3. Optimization of Response Surface Experimental Results

The quadratic polynomial regression model was optimally solved using Design Expert 8.0.6 software, and nine scenarios were obtained. The optimal values of the final concentrations of oligomeric mannose, maltose, and sorbitol in the scenarios were 0.46%, 0.45%, and 0.05% (*w*/*v*), respectively. The theoretical minimum particle size value of ABC-NPs lyophilized powder prepared with the above concentrations of lyophilized protectant was 357 nm after re-dissolution.

To check the reliability of the results, three validation experiments were conducted using the optimal conditions optimized by the response surface. The experimental results were 472 nm, and the ratio of the re-solubilized particle size to the initial particle size S_a_/S_b_ of ABC-NPs suspension was 1.32. The values of the experimental results were close to the theoretical values, indicating that the model can better predict the protective effect of lyophilized protectant concentration on ABC-NPs.

In our research, the natural polymers such as polysaccharides have the advantages of biodegradability, safety, low toxicity, and good stability, and they are in ionic form in water and can spontaneously bind to proteins. In addition, the protein–polysaccharide system can prevent the structure and properties of biochemical drugs from being destroyed, and nanoparticles for nanocarriers can achieve slow and controlled release of drugs, so it has become a central tool for nanodrug delivery research [42].

Maltose and sorbitol are commonly used as lyophilization protectants [43,44,45,46]. Our experiments demonstrated that maltose and sorbitol can effectively protect ABC-NPs with J-type astaxanthin aggregates which have various bioactive function [47,48], presumably because their surfaces contain a large number of hydrogen bonds that can provide support for nanoparticles and have a high glass transition temperature [15,16,20]. Oligomannose is not commonly seen in previous studies. This experiment verified that oligomannose has a practical protective effect on nanoparticles in the protein–polysaccharide system.The samples after adding the compound lyophilized protectant were pink in color; the samples were fluffy and completely dissolved within 10 s after shaking with water, indicating that the effect of the compound protectant was significantly better than that of the single protectant.In other international studies, the particle size compounding ratio is generally between 1.02 and 1.52 [49,50,51,52,53], while our particle size compounding ratio is 1.32. This indicates that our results are at a stable level internationally and can provide theoretical support for future studies on protein–polysaccharide systems and lyophilized protectants.

## 4. Conclusions

Freeze drying is an effective way to improve the long-term physical stability of nanosuspensions during preservation. Freeze-drying process agglomerates the particles, whereas the addition of cryoprotectants and ultrasonication procedure can reduce the particle size. In this study, the mass-produced homogeneous ABC-NPs was successfully carried out, and the particle sizes were about 320–360 nm, the PDIs were all below 0.3, and the Zeta potentials were all around +15 mV. The ABC-NPs nano-suspension was freeze-dried to obtain a pink fluffy lyophilized powder with a redissolving particle size about 550–680 nm. The optimal ratio of lyophilized protectants was obtained through response surface methods as 0.46% oligomannose, 0.44% maltose, and 0.05% sorbitol (*w*/*v*). Under these conditions, the re-dissolved particle size of ABC-NPs lyophilized powder was 472 nm and the Sa/Sb value was 1.32. The color of lyophilized powder remained unchanged and the structure was looser, indicating that the structure and activity of astaxanthin nanoparticles were well preserved. The Sa/Sb value is an important factor in evaluating the effectiveness of lyophilized protectants.1) These results of this study provide theoretical foundation and academic references for the long-term preservation, deep processing, and resource utilization of ABC-NPs, and also expand the applications of nanocarriers in food, pharmaceutical, and biological fields.

## Figures and Tables

**Table 1:** Box–Behnken factorial design (BBD) of three variables and response values.

No.	Variables			Size (nm)	PDI
	A: Concentrion of Maltose (%)	B: Concentrion of Oligomeric Mannose (%)	C: Concentrion of Sorbitol (%)		
1	−1	−1	0	532	0.29
2	1	−1	0	503	0.28
3	−1	1	0	475	0.26
4	1	1	0	466	0.24
5	−1	0	−1	493	0.29
6	1	0	−1	463	0.31
7	−1	0	1	469	0.29
8	1	0	1	468	0.25
9	0	−1	−1	479	0.25
10	0	1	−1	444	0.29
11	0	−1	1	493	0.26
12	0	1	1	450	0.26
13	0	0	0	431	0.24
14	0	0	0	448	0.26
15	0	0	0	465	0.29
16	0	0	0	450	0.31
17	0	0	0	435	0.32

**Table 2:** ANOVA for response surface quadratic models.

Source	Sum of Squares	d_f_	Mean Square	F Value	Prob > F	
Model	9903.19	9	1100.35	8.06	<0.005	*
A-maltose	595.12	1	595.12	4.36	0.0751	
B-oligomeric mannose	3698.00	1	3698.00	27.10	<0.005	*
C-sorbitol	0.12	1	0.12	9.162 × 10^−4^	0.9767	
AB	100.0210.250	1	100.0210.250	0.73	0.4203	
AC	16.00	1	16.00	1.54	0.2544	
BC	3178.42	1	3178.42	0.12	0.7421	
A^2^	1808.53	1	1808.53	23.3	<0.005	*
B^2^	2.632 × 10^−3^	1	2.632 × 10^−3^	13.26	0.0083	
C^2^	955.05	1	136.44	1.929 × 10^−5^	0.9966	
Residual	228.25	7	76.08			
Lack of fit	726.80	3	76.08	0.42	0.7498	not significant
Pure error	726.80	4	181.70			
Cor total	10,858.24	16				

* *p* < 0.01

## Data Availability

All data were authentic.
